# Multifunctional Nanotherapeutics for the Treatment of neuroAIDS in Drug Abusers

**DOI:** 10.1038/s41598-018-31285-w

**Published:** 2018-08-28

**Authors:** Rahul Dev Jayant, Sneham Tiwari, Venkata Atluri, Ajeet Kaushik, Asahi Tomitaka, Adriana Yndart, Luis Colon-Perez, Marcelo Febo, Madhavan Nair

**Affiliations:** 1 Institute of NeuroImmune Pharmacology, Center for Personalized Nanomedicine, Department of Immunology and Nano-Medicine, Herbert Wertheim College of Medicine, Florida International University, Miami, FL 33199 USA; 2 Department of Psychiatry, The McKnight Brain Institute, University of Florida, Gainesville, FL 33610 USA

## Abstract

HIV and substance abuse plays an important role in infection and disease progression. Further, the presence of persistent viral CNS reservoirs makes the complete eradication difficult. Thus, neutralizing the drug of abuse effect on HIV-1 infectivity and elimination of latently infected cells is a priority. The development of a multi-component [antiretroviral drugs (ARV), latency reactivating agents (LRA) and drug abuse antagonist (AT)] sustained release nanoformulation targeting the CNS can overcome the issues of HIV-1 cure and will help in improving the drug adherence. The novel magneto-liposomal nanoformulation (NF) was developed to load different types of drugs (LRAs, ARVs, and Meth AT) and evaluated for *in-vitro and in-vivo* BBB transmigration and antiviral efficacy in primary CNS cells. We established the HIV-1 latency model using human astrocyte cells (HA) and optimized the dose of LRA for latency reversal, Meth AT in *in-vitro* cell culture system. Further, PEGylated magneto-liposomal NF was developed, characterized for size, shape, drug loading and BBB transport *in-vitro*. Results showed that drug released in a sustained manner up to 10 days and able to reduce the HIV-1 infectivity up to ~40–50% (>200 pg/mL to <100 pg/mL) continuously using single NF treatment ± Meth treatment *in-vitro*. The magnetic treatment (0.8 T) was able to transport (15.8% ± 5.5%) NF effectively without inducing any toxic effects due to NF presence in the brain. Thus, our approach and result showed a way to eradicate HIV-1 reservoirs from the CNS and possibility to improve the therapeutic adherence to drugs in drug abusing (Meth) population. In conclusion, the developed NF can provide a better approach for the HIV-1 cure and a foundation for future HIV-1 purging strategies from the CNS using nanotechnology platform.

## Introduction

**Figure 1:**
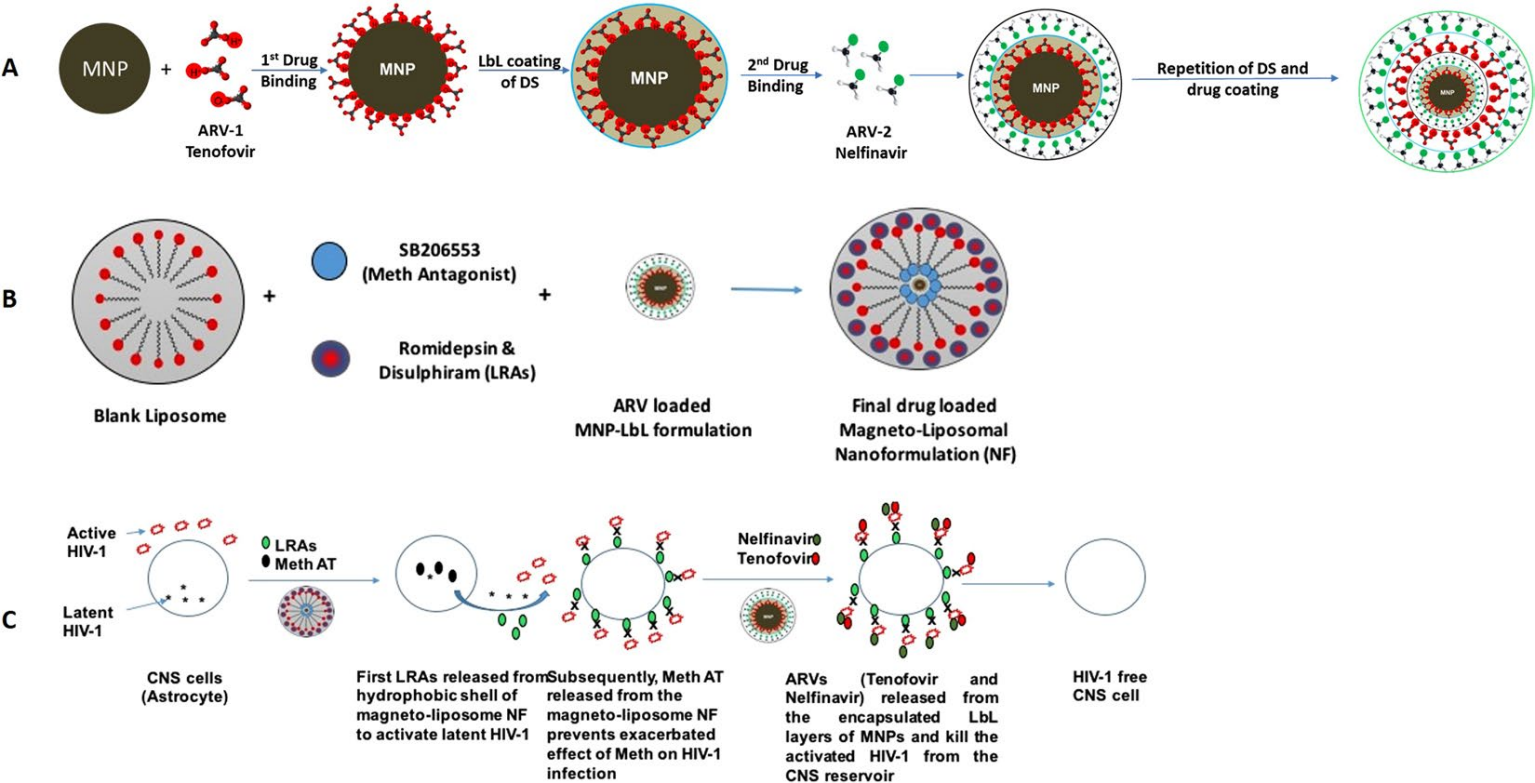
Schematic representation of the development of magneto-liposome NF for drug delivery across the BBB: (**A**) MNP-LbL assembly of drugs; (**B**) Magneto-liposome NF preparation; (**C**) Proposed mechanism of action of NF.

HIV-1 infection remains incurable due to the presence of quiescent, replication-competent provirus within a long-lived population of memory T cells, which are capable of reigniting new rounds of infection if highly active antiretroviral treatment (HAART) therapy is interrupted. While an undetectable viral load is achieved in most HAART treated patients; latent viral reservoirs continue to permanently harbor HIV proviral DNA in resting memory CD4+ T cells^1–3^. The challenges of lifelong HAART include drug toxicity, the development of drug resistance, adverse drug interactions, stringent treatment adherence and high costs of treatment^4^. Along with peripheral reservoir, the central nervous system (CNS) is also a major target of HIV and is responsible for a number of neurologic disorders including neuroAIDS^5–7^. HIV infection of the CNS is associated with the development of asymptomatic neurocognitive impairment, HIV-associated mild neurocognitive disorder (HAND), and HIV-associated dementia (HAD) that manifest as a clinical syndrome of cognitive, motor, and behavioral dysfunction^8^. Further, neuroinflammation associated with HAND appears to be exacerbated by drugs of abuse, as demonstrated by brain autopsy studies revealing a higher prevalence of HIV encephalitis (microglia activation, the presence of multinucleated giant cells, and blood–brain barrier (BBB) disruption) in drug-abusing HIV-1 positive individuals in comparison with non-abusing HIV-1 positive controls^9,10^. Methamphetamine (Meth- 2^nd^ most abused drug in the USA) intake promotes further progression of HIV-1 disease and increases the viral replication as shown by several previous *in-vivo* studies^11^. Also, clinical studies in humans suggest that current meth users taking HAART to treat HIV-1 may be at greater risk of developing AIDS than non-users, possibly as a result of poor medication adherence^12,13^. Meth abusers with HIV-1 also have shown greater neuronal injury and cognitive impairment due to HIV-1, compared with those who do not abuse the drug^14^. These neurological complications are most likely due to the early penetration of HIV-1 into the CNS *via* infected immune cells such as CD4+ T lymphocytes, dendritic cells, monocytes, and macrophages, which are all cellular reservoirs of HIV-1^2,15,16^. There are numerous mechanisms propositioned for HIV-1 latency i.e. restriction factors *via* cellular pathways, RNA interference, integration of the proviral DNA in transcriptionally dormant site, Tat-activated elongation factor (P-TEFb), histone modifications or unavailability of cellular transcription factors like NF-kB that act as co-activators of the HIV-LTR^17,18^. HIV post-integration latency is mainly due to transcriptional silencing that involves chromatin reorganization^19^. Current HAART therapy lacks the component capable of reactivating latent viral infection. Thus, latent viral reactivation component is essential along with HAART to purge the virus from compartmentalized latent viral CNS reservoirs. As per previous reports, latent HIV responds to T-cell activation signals^20^. T-cell activation strategies include treatment with pro-inflammatory cytokines such as IL-6, TNF-a, IL-2, and in monocyte/macrophages IFN-c^21^. However, these combinations lead to T-cell reduction and ricochet in viral load when HAART is removed. Due to the low frequency and different type of latently infected cells in CNS reservoir, a single or combination of different latency reactivating agents (LRAs) may be useful in breaking the latency in CNS. Clinical trials in patients on HAART are ongoing with different class of LRA’s e.g. Disulfiram [inhibitor of acetaldehyde dehydrogenase and reactivate HIV-1 *via* depletion of the phosphatase and tensin homolog-PTEN inhibitor]^1^, vorinostat, romidepsin and panobinostat [Histone deacetylase inhibitors (HDACi)]^1^ and Phorbol esters (PMA), Prostratin and Bryostatin-1 [Protein kinase C (PKC) agonists]^17,22^ for reversing the latency in peripheral reservoirs but none of them have been reported or explored in latent CNS reservoir eradication. The reason for their ineffectiveness in the CNS is due to their non-penetrability of LRAs across blood-brain-barrier (BBB). Our group already has shown the delivery of non-BBB penetrable anti-HIV drugs and neuroprotective agents across BBB using noninvasive external magnetic force and demonstrated that this target specific (CNS) strategy can be used for the treatment of neuroAIDS *via* application of magnetic nanocarriers^6^. Thus, the aim of this work was to develop and standardize the experimental latent HIV-1 reservoir model using primary CNS cells (e.g. astrocyte), and secondly, to test the delivery efficiency and therapeutic evaluation of sustained release liposomal-magnetic nanoformulation (NF) loaded with LRA’s for latency breaking, anti-HIV drugs to prevent the HIV-1 replication after activation or active infection, Meth antagonist to counter attack Meth abuse and its associated HIV-1 infected. Additionally, we are studying the effects of drug abuse (Meth), on the latency development and its correlation and effect on the efficacy of the NF. To our knowledge, this is the first comprehensive attempt to encapsulate LbL-assembled MNP in PEGylated liposome (Magneto-liposome) for the targeted delivery of drug combination across the BBB in a non-invasive manner (by magnetic force) packed in a single nanoformulation for the treatment of neuroAIDS. We also have shown the hypothetical approach for the nanoformulation efficacy for the complete eradication of HIV-1 reservoir form the CNS cells as shown in Fig. 1.

## Materials and Methods

### Cell culture and reagents

Primary human astrocytes (HA) were purchased from ScienCell Research laboratories (Carlsbad, CA; Cat. #1800-5) and grown in astrocyte medium purchased from ScienCell laboratories (Cat. #1801) containing 2% of fetal bovine serum (ScienCell Cat. # 0010), astrocyte growth supplement (ScienCell Cat. #1852) and penicillin/streptomycin (ScienCell Cat. #0503). HIV-1_Ba-L_ (clade B) (NIH AIDS Reagent Program Cat. #510) was obtained through AIDS Research and Reference Reagent Program, Division of AIDS, NIAID, NIH.

### HIV-1 infection of HA cells and *in-vitro* latency model development and evaluation of latency reactivating agents

The HA were infected with HIV-1 using the previously described protocol^6^ with slight modifications. Briefly, HA (0.1 × 10^6^ cells) cells were cultured overnight in 6 well plates using astrocyte medium. The cells were activated by treating with polybrene (10 µg/ml) for 6–7 hrs before the infection. The cells were infected with TCID_50_ of HIV-1 clade B virus for a total of 25 days under same experimental conditions. For the latency development experiment, on 3, 6, 9 and 14 day fresh medium (500 µL) was added and every day the supernatant (500 µL) and cells were isolated till 16 days of the infection period. On 14 day, a dose of different LRAs (e.g. Bryostatin, Vorinostat, Romidepsin, Disulfiram, PMA) were optimized using different concentrations (48 hr treatment). Collected medium was used for the p24 antigen estimation using an ELISA kit (ZeptoMetrix Corp. Cat # 0801200) and RNA was isolated from retrieved cells for LTR estimation by LTR-qPCR array. Controls cells (without clade B infection) were included in the set-up of all experiments.

### mRNA isolation

Pellet from collected cell samples after each day treatments were suspended in lysis buffer and was used for the mRNA isolation using illustra triplePrep Kit (GE Healthcare Life Sciences, UK; Cat# 28-9425-44) and on-column DNase treatment step was also performed in the procedure. The purity of the RNA was measured by microspot RNA reader (Synergy HT Multi-Mode Microplate Reader from BioTek, US) and RNAs with an OD_260nm_/_280nm_ absorbance ratio of at least 2.0 were used. This mRNA was used for the long terminal repeat (LTR)-gene expression using RT-qPCR to measure the HIV infectivity.

### QPCR of viral transcripts

The cDNA for mRNA isolated from all controls and test groups (day-10 or LRA treated) was synthesized using the high-capacity reverse transcriptase cDNA kit (Applied Biosystems, Cat # 4368814) to perform qRT-PCR by Taqman gene expression assay as described by manufacturer’s protocols. A quantitative HIV-1 DNA protocol (LTR real-time PCR) was used to analyze the viral transcripts in HIV infected HA cells alone or LRA’s treated samples. The following previously published^23^ primers and probes were used: LTR U5/R-sense-5′-GGCTAACTAGGGAACCCACTG-3′ and antisense-5′-CTGCTAGAGATTTTCCACACTGAC-3′, probe 5′-FAM-TGTGTGCCCGTCTGTTGTGTG-TAMRA-3′. Values were normalized against GAPDH.

### Preparation of PEGylated magneto-liposomes nanoformulation (NF), characterization and transferrin conjugation

NF was prepared as per our previously published protocol^24^ using optimized 7:2:0.5 molar ratios of Egg Phosphocholine (PC), Cholesterol (CL), and mPEG2000-DSPE. LRAs (Disulphiram and Romidepsin) were dissolved in 9:1 ratio to ethanol: chloroform and loaded into organic lipid part i.e. mPEG2000-DSPE ratios (w/w), while (MNP + Nel + Teno) + SB-206553 (Meth antagonist) was dissolved in PBS and loaded in the aqueous part. Unencapsulated drugs/MNP was removed by centrifugation at 15000 rpm for 30 minutes and drug loaded liposomes were obtained and quantified for loading. Finally, transferrin was conjugated to liposome by mixing 0.5 mg transferrin into 200 µL of prepared NF using EDC-NHS method as per published protocol^25^. The liposomal NF was characterized for size and shape, surface charge, % drug loading on liposome surface. The percentage encapsulation efficiency and quantitative analysis of encapsulated MNPs loaded in liposomes was also calculated as described by us previously^24^.

### *In-vitro* blood-brain-barrier (BBB) preparation, validation and NF transmigration assay

Primary human brain microvascular endothelial cells (HBMEC) and human astrocyte (HA) cells were cultivated as per provider’s recommendations. The BBB model was established as described earlier by Persidsky *et al*.^26^ and modified by us^6^. More details of the method are provided under the supplemental method section.

### Evaluation of *in-vitro* efficacy of NF in HIV-1 infected cells

HIV-1 infected human primary astrocytes (HA) were treated with NF and checked for their *in-vitro* latency breaking efficacy and antiviral efficacy using p24 antigen assay^6^ in the presence and absence of Meth. Overall efficacy of NF was compared with single component formulations.

### *In-vitro* cytotoxicity analysis of NF

Cytotoxicity of MNP alone, drug loaded LbL-MNP, overall NF was assessed by MTT cell viability assay using assay kit (Catalog # 11465007-001, USA)^27^. HA’s were seeded in 96-well culture plates at a density of 10,000 cells per well. After 24 hr of incubation at 37 °C, the culture medium was replaced with 100 µl fresh media containing different concentrations of all the samples along with controls (50–250 µg/mL). 20 µL of MTT solution (5 mg/ml in PBS) was added into each well after 24 hr post-treatment and incubated at 37 °C for 2 hr. Finally, 100 µL of stop solution (20% SDS in 50% Dimethyl Formamide) was added into each well and absorbance was recorded at 550 nm by the microplate reader (Synergy HT, Multi-mode microplate reader, BioTek Instrument, Inc., Winooski, Vermont, USA) for 24 and 48 hr NF treated samples.

### Animals

Mice experiments were approved by the Institutional Animal Care and Use Committee (IACUC) of University of Florida (UF) and were in accordance with the National Institutes of Health Guide for the Care and Use of Laboratory Animals. Male BALB-C mice (2 months old, wt. 25 ± 3.5 gm, Charles River Laboratories, Raleigh, NC, USA) were used. Bedding, nesting material, food, and water were provided *ad libitum*. Ambient temperature was controlled at 20–22 °C with constant 12-hr light/12-hr dark cycles.

### MRI and Image analysis

For NF biodistribution and BBB transport, Magnex Scientific 4.7 Tesla MR scanner was used for performing high-resolution imaging for all the MRI studies. The T2 and T1 relaxometry pulse sequences were run on a Varian VnmrJ 3.1 console. The T2 and T1 relaxation rates were assessed separately using MRI on a series of phantom tubes filled with a mixture of Agarose with varying concentrations of magnetic nanoparticle (0–100 µM). More details about the image capturing are provided under the supplemental method section.

### Statistical analysis

The results are expressed as the mean ± S.D. of three to five independent experiments for each graph. Statistical analysis was performed with Prism software (Graph Pad Software, San Diego, California, USA) using and differences between two groups were calculated using two-tailed student’s t-test or using non-parametric tests followed by one-way analysis of variance for pairwise comparisons and were considered significant when *p ≤ 0.05 and **p < 0.01 or ***P < 0.001; ****P < 0.0001.

## Results

### Establishment of HIV-1 latency model using CNS cell

**Figure 2:**
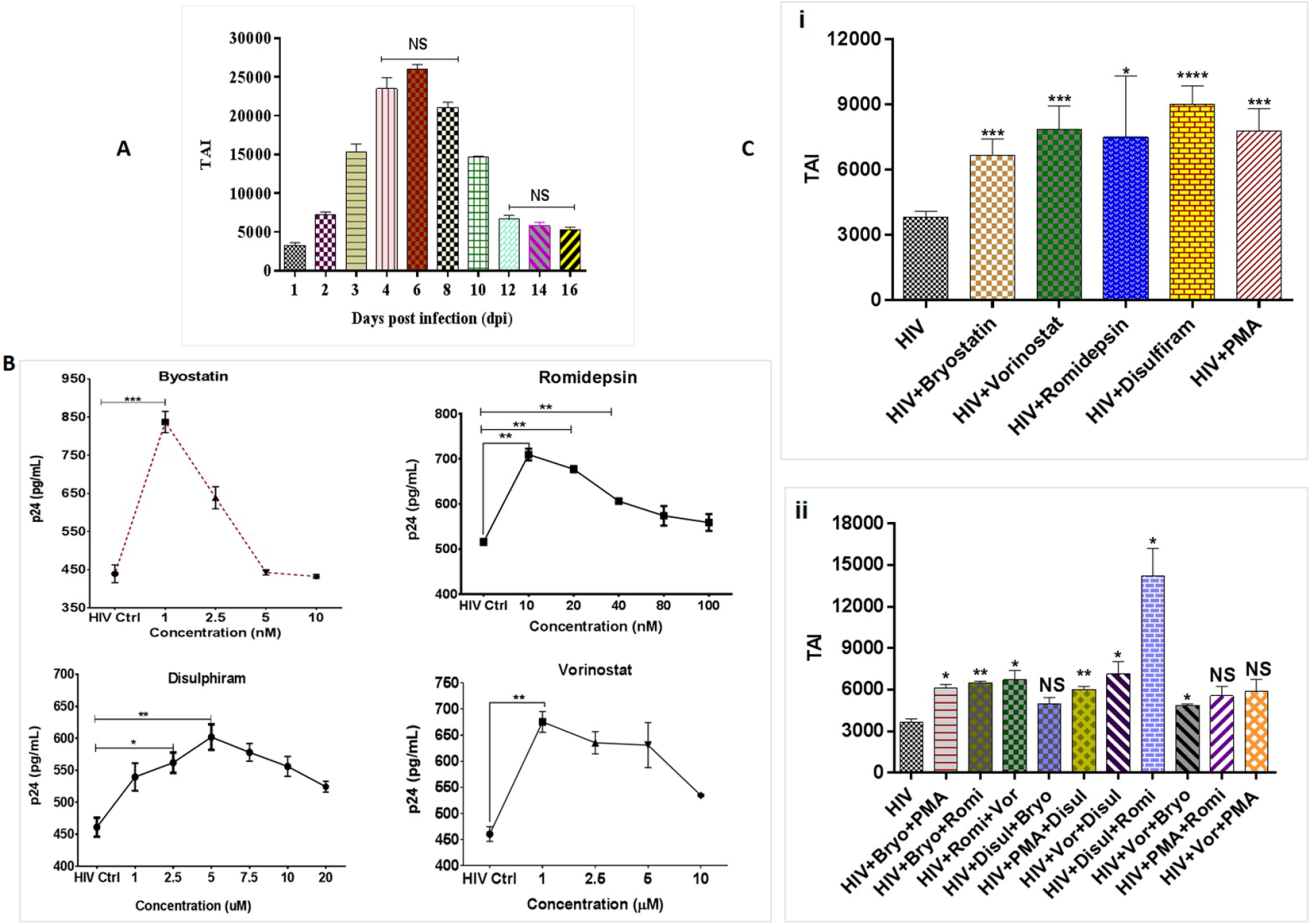
(**A)** Development of latent HA model; (**B**) Dose optimization of different LRA agents; (**C**) Effects of latency reversing agents on provirus quiescence in HA populations (14 dpi), latent HIV cells were treated with optimized dose of (a) Bryostatin: 1 nm; (b) Vorinostat: 1 µM; (c) Romidepsin: 10 nm; (d) Disulphiram: 5 µM; (e) PMA: 5 nm (positive control) (i) single agent treatment (ii) combination treatment and analyzed for HIV-1 expression for 48 hrs in comparison to untreated controls. Results demonstrate differential effects of LRAs on latent HIV-1 in HA populations. Data are from one infection experiment for each latent cell population, with columns indicating mean values for each time point. Standard errors are shown for the mean of triplicate samples.

To generate an experimental CNS model for HIV-1 latency, proliferating HA cell populations were infected with HIV-1 (clade-B) and monitored for quantities of HIV-1 p24 antigen and LTR expression for over 16 days post infection (dpi). Results showed that LTR expression increases up to day 6, cells go into dormant phase from day 12 (dpi) onwards, and reach the plateau by day 16 (dpi), as no significant differences in LTR levels were observed from day 12 till day 16 (Fig. 2A). Thus, results confirm the generation of HA cells with latent HIV provirus. For all the latency related experiments, day 14 (dpi) cell cultures was used for further dose optimization and NF efficacy studies.

### Dose optimization of LRAs for HIV-1 latency reversal

To investigate reactivation of HIV-1 latency, HA’s were exposed (48 hrs) to different classes of LRAs i.e. vorinostat and romidepsin (HDACi); bryostatin (PKC activator) and disulphiram (PTEN agonists) at different concentrations to reactivate HIV-1 in latently infected HA (14 dpi) as shown in Fig. 2B. PMA, DMSO, and cells without HIV-1 infection were used as a positive, vehicle and negative controls, respectively. The concentration range of each LRA was selected based on the previous studies by other groups for latency breaking for peripheral or CNS cells. Results showed that a concentration of 1 nM for bryostatin, 10 nM for romidepsin, 1 µM for vorinostat, 5 µM for disulphiram as shown in Fig. 2B and 5 nM for PMA was optimum with respect to cellular toxicity (Supplementary Fig. S1) and corresponding p24 production in the HIV treated cells. All the optimized concentrations of each LRA showed significant reactivation (Fig. 2C-i) in HIV-1 treated samples. Further, we tested the possible synergistic effect of all the LRAs, where disulphiram and romidepsin combination showed most significant results for LTR activity and showed differential effects of known LRAs on latent HIV-1 cells (Fig. 2C-ii). All further experiments, with a single agent or a combination of LRAs, were performed with the above mentioned optimized doses. All the optimized concentrations showed positive cell viability (Supplementary Fig. S3) as assessed by MTT cytotoxicity assay after 48 hrs of treatment.

### Development and characterization of NF

**Figure 3:**
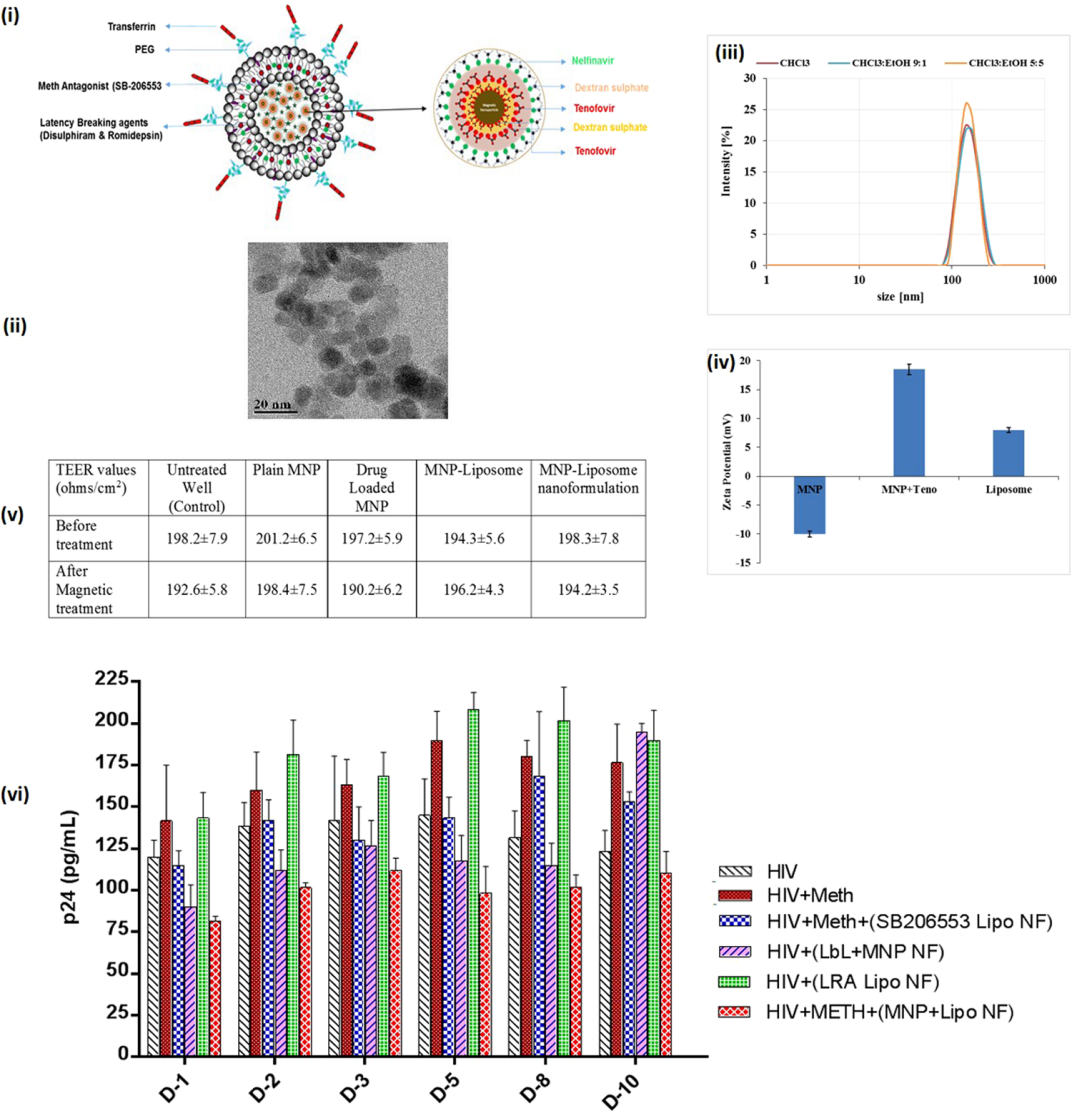
Nanoformulation (NF) design, characterizations and efficacy evaluation: (**i**) Schematic representation of nanoformulation design; (**ii**) Transmission electron microscopy of MNPs, average size range: 10 ± 3 nm (**iii**) hydrodynamic size of magneto-liposome in different solvent condition; (**iv**) Zeta potential analysis of blank MNP, 1BL coated drug loaded MNP and drug loaded magneto-liposome in PBS; (**v**) Transendothelial electrical resistance (TEER) values of the *in-vitro* BBB model before and after treatment of NF in the presence and absence of external magnetic force; (**vi**) NF efficacy in HIV-1 infected HA in presence of Meth: HA (1 × 10^5^ cells) were grown in 6 well culture plates and cells were infected with 20 ng of HIV-1 clade B for overnight. Unbound virus was washed with PBS and cell were infected for 14 days so that cell goes into Latent phage. On the 15 day of infection, optimized Meth (25 µM) was added to cells and treated every day for next 10 days (total 25 days of HIV infection). Drug-loaded NF (100 µg/ml) was added only once (on the16 day) to the respective wells and effect of NF on HIV infection ± Meth levels were measured by using the p24 ELISA. Results were analyzed with respect to HIV-1 v/s HIV-1+ Meth v/s NF treatment (*p ≤ 0.05; **p ≤ 0.01; ***p ≤ 0.001; ****p ≤ 0.0001; NS-Not Significant); Standard errors are shown for the mean of triplicate samples. TEM-Magnification 200 K, Scale bar –100 nm.

Schematic representation of the development of LbL assembled drug loaded MNPs encapsulated within PEGylated liposome NF loaded with antiHIV drugs, Meth antagonist and LRAs is shown in Fig. 3(i). The ultra-small Fe_3_O_4_ magnetic nanoparticles (MNPs) were prepared and characterized for their size, shape, and drug binding efficiency. Transmission electron microscopy (TEM) demonstrates that the average size of MNPs were approximately 10 ± 3 nm and uniform in size and shape as shown in Fig. 3(ii). The loading efficiency of the 1^st^ layer of Tenofovir (35 ± 3.5%), 2^nd^ layer (28 ± 4.5%) and Nelfinavir (25 ± 2%) were obtained in LbL assembled MNPs. LbL assembly deposition was confirmed using zeta (ζ) potential analysis. Results showed that the ζ-potential value for uncoated MNPs coated with Tenofovir was +18.5 ± 3.5 mV, which reversed upon adsorption of dextran sulfate (DS) coating to −22.3 ± 1.5 mV. Further, to encapsulate the hydrophobic LRAs in the liposome, different solvent combinations (Chloroform or Chloroform to Ethanol in 90:10 or 50:50 ratios) were used for the synthesis of PEGylated liposomes. Dynamic light scattering (DLS) analysis was used for hydrodynamic size analysis (Fig. 3-iii). Reversal of charge due to DS coating helps in loading more drugs on to the surface of the nanoparticle, which is not possible without the LbL technique (Fig. 3-iv). Table 1 highlights the characterization of all different type of nanoformulations with respect to drug loading on MNPs, MNPs loading in the liposome, hydrodynamic size and surface charge respectively. For the final assembled magneto-liposome nanoformulation (NF) development, we used Chloroform to Ethanol in 50:50 ratio.

**Table 1:** Characterization of MNPs, Drug loading and Magneto-liposomal formulations.

Formulation type	Drug loading (%age)	MNP encapsulation efficiency	Hydrodynamic Size (nm)	Zeta Potential (mV)
MNP	—	—	18 ± 2.0	−10 ± 2.5
MNP + Tenofovir (1^st^ Layer)	35 ± 3.5%	—	18.5 ± 3.5	18.5 ± 3.5
MNP + Tenofovir (2^nd^ Layer)	28 ± 3.5%	—	18.5 ± 1.5	18.0 ± 2.0
MNP + Nelfinavir	25 ± 2.0%	—	18.5 ± 2.0	16.5 ± 4.5
Blank Liposome	—	—	340 ± 12.5	7.8 ± 1.5
Liposome + unloaded MNP	—	20 ± 4.0%	360 ± 14.0	7.8 ± 2.5
Liposome + LRAs	—	18.0 ± 4.0%	345 ± 10.5	8.5 ± 2.2
Liposome + Drug loaded MNP	—	16.5 ± 3.5%	380 ± 12.8	8.3 ± 2.9
Final NF (Liposome + MNPs + Drugs)	—	18.0 ± 4.0%	410.0 ± 15.0	8.2 ± 2.5

(MNP Amount: 1 mg; Tenofovir- 1 mg/ml; Nelfinavir- 1 mg/ml, Liposome- 1 mg/mL, Romidepsin- 1 mg/ml, Disulphiram -1 mg/ml.

The BBB transmigration of the plain MNPs; MNPs-drug-loaded and NF (Magneto-liposome nanoformulation) were evaluated using an *in-vitro* BBB model composed of primary human cells (primary human brain microvascular endothelial cells and primary astrocytes). The intactness of the developed BBB model was estimated *via* measuring TEER. The TEER values of all treatment groups were similar to standard 190 ± 10 ohms/cm^2^ when compared with respect to all different nanoformulations (Fig. 3-v). Thus, proving that the transmigration of MNPs or NF under external magnetic field does not affect the BBB integrity. Further, nanoformulations transmigration ability to cross BBB was confirmed by measuring the amount of iron, on application of the external magnetic field. Results showed that 35 ± 5% of plain MNPs versus 15.8% ± 5.5% of final magneto-liposome NF cross the BBB in the presence of 0.8 T magnetic field for 3 hours. Finally, to overcome the exacerbated effects of drugs of abuse (Meth) on HIV-1 infection, the efficacy of MNP-antiHIV drugs NF, Liposome-LRA NF and magneto-liposome NF was evaluated in HIV-1 infected primary HA cells. The NF efficacy was determined *via* quantification of p24 antigen level in latently infected HA cells +/− Meth. After 14 dpi, HAs were treated every day with the optimized dose of Meth-25 µM (Supplementary Fig. S2) from Day 15 till 25 (Total NF treatment = 10 days). Different NFs were added (100 μg/mL) and the supernatant was collected every day till 10 days to study the latency breaking ability of LRAs, antagonist efficacy of meth antagonist and antiviral efficacy of antiviral drugs for the NF efficacy. ELISA p24 assay results (Fig. 3-vi) showed a significant increase in the p24 levels upon Meth treatment (120 to 200 pg/ml) when compared with HIV only control cells (100 to 140 pg/ml) over the entire treatment periods. Results also showed that meth antagonist (SB206553) release attenuates the additive viral activity of HIV-1 against Meth. The sustained-release ability of the NF was evaluated over a treatment period of 10 days and data showed ~ 40–50% (>200 pg/mL to <100 pg/mL) constant reduction in viral load over the entire period of NF treatment. Thus, confirming the capability of the developed NF to attenuate the effect of drug abuse and suppressHIV-1 replication concomitantly and in a sustained manner.

### *In-vitro* cell uptake and cytotoxicity evaluation of NF

**Figure 4:**
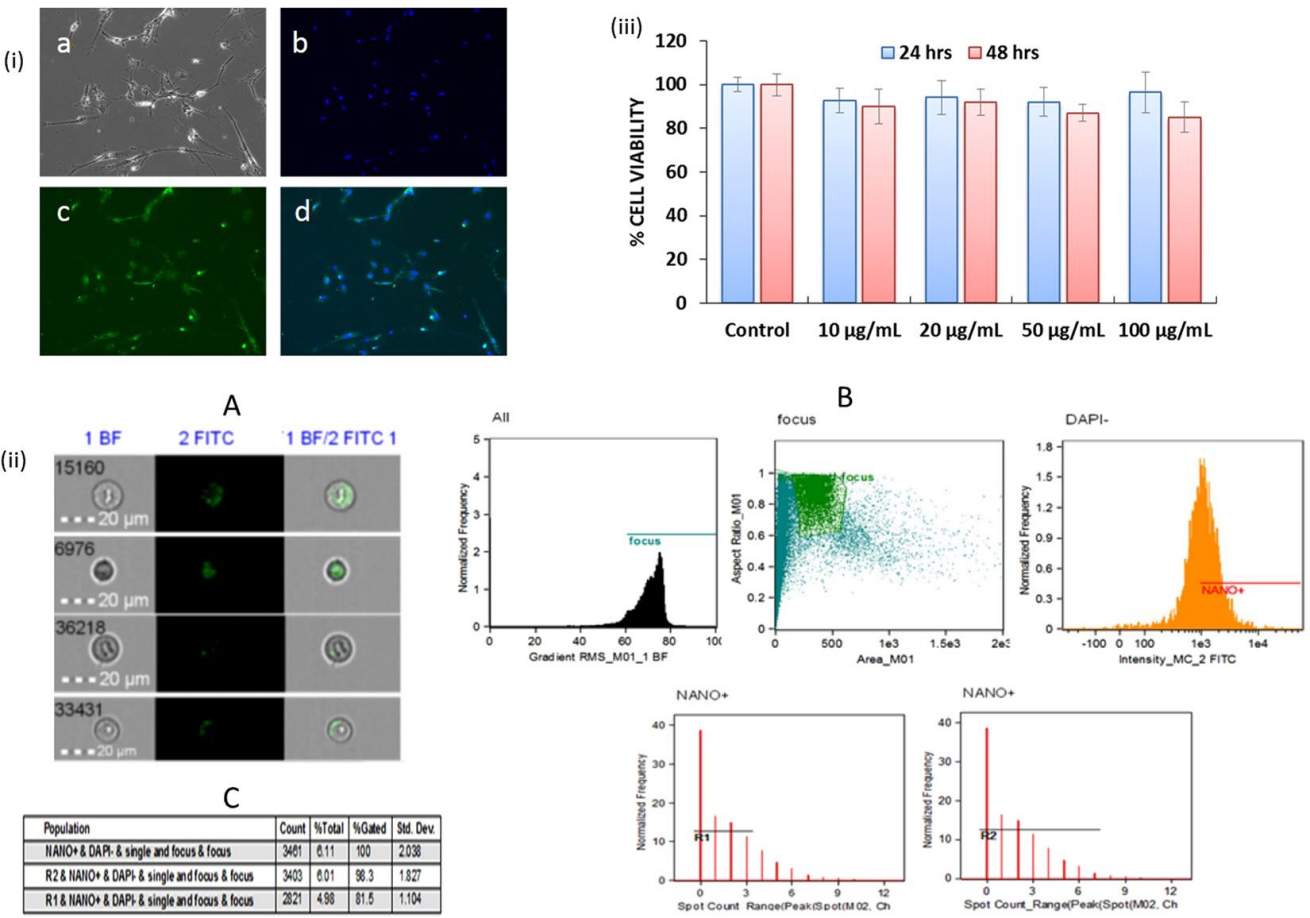
(i) Qualitative analysis of cellular uptake of FITC-tagged NF in HA by fluorescence microscopy: FITC-tagged NF (concentration- 100 μg/mL) in HA after 3 hours of treatment. (a) Control cell bright field image; (b) Cell nuclei stained with DAPI (blue); (c) FITC tagged drug loaded NF (green); (d) Composite image show green and blue fluorescence inside the cells, confirm the cellular uptake of the NF. Images taken at 10X magnification using Fluorescence microscopy (Zeiss, Wetzlar, Germany); (ii) Quantitative analysis of FITC-tagged NF in HA by flow cytometry: (**A**) Cell images of internalized and surface-bound NF in HA: Representative images from the Amnis ImageStreamX; (**B**) Schematic of the gating strategy utilized to determine internalized versus surface bound nanoparticles: To gate on cells in-focus, the IDEAS feature *Gradient RMS* of the bright field image is plotted in a histogram, further Green fluorescence FITC positive cells were selected by gating on the cells with high *Max Pixel* values and *Intensity* in the green fluorescence channel and DAPI negatives. Also, cells with internalized NF were selected by choosing the cell population with an internalization score equal to or greater than 0.3. Representative images are presented for each gate shown; (**C**) Spot analysis statistics: Cells in the “internalized” gate were further characterized based on the number of spots because there were some cells with background staining that were counted as internalized but had a spot value of zero as shown in table for different region R1 (0–3 counts) and R2 (0–8 counts); (iii) *In-vitro* cytotoxicity of NF: Results show the percentage of cells viability after treatment with different NF concentrations (10, 20, 50 and 100 µg/mL). HA (1 × 10^5^ cells) were grown in 6 well culture plates and cells were infected with 20 ng of HIV-1 for overnight. The uninfected virus was washed and cells were infected for a total of 14 days. On 15 day of infection, NF was added at different concentrations to the respective wells and incubated for 24 and 48 h respectively. After total 16^th^ and 17^th^ day of post-infection (dpi), cell viability was measured by using the MTT assay (*p ≤ 0.05; NS-Not Significant).

The qualitative and quantitative analysis of NF with respect to the cellular uptake efficiency was examined using the different concentrations (50, 100, 150 and 200 µg/ml) in latently infected HA cells. For qualitative uptake studies, NF was tagged with FITC for facilitating the observation *via* fluorescence microscopy, and results (Fig. 4-i) showed NF (representative image of 100 µg/ml concentration treated sample) was taken up by the HA very efficiently. Our results also showed the concentration-dependent increased in the percentage of cells positive for FITC-tagged NF, as the NF concentration increased from 10 to 100 μg/mL after 3 hrs of treatment (Data not shown).

Further to understand the process of NF uptake and quantitative analysis, we used single cell flow cytometry to investigate cell uptake kinetics and characterized them for internalization process using HIV-1 infected HA cells. We performed the NF uptake studies with respect to time and dose, the result showed (Fig. 4-ii-A) increase in spots observed in bright field images, which proved higher cell uptake. Further, we did not observe higher cell uptake with longer incubation times (data not shown) and saturation was observed around 24 hours. When uptake efficiency was calculated (total amount of NF taken up by cells divided by the amount of NF added to cells), we found that, although spot count leveled off after 6 hours, NF continued to be taken up by the cells as late as 24 hours. The IDEAS software internalization wizard also verified higher fluorescence intensity inside cells (FITC) as compared to the fluorescence intensity of the entire cells, suggesting that NF gets internalized rather than remaining surface bound. Representative images are presented for each gate (Fig. 4ii-B). Statistical analysis shows that (Fig. 4ii-C), 98.3% and 81.5% cells get internalized for region R-1 and R-2 respectively when gated for internalization. Finally, to evaluate the *in-vitro* cytotoxicity of the NF, we performed MTT cytotoxicity assay on HIV-1 infected HA cells. The results show (Fig. 4-iii) that treatments with different concentrations (10–100 µg/ml) of NF did not cause cytotoxicity, as the percentage of viable cells, was similar to untreated control up to 48 hrs of treatment. Thus, signifying that all the tested doses of magneto-liposome NF were nontoxic and developed NF will not have any safety concerns for *in-vivo* application.

### *In-vivo* MRI and NF toxicity studies

**Figure 5:**
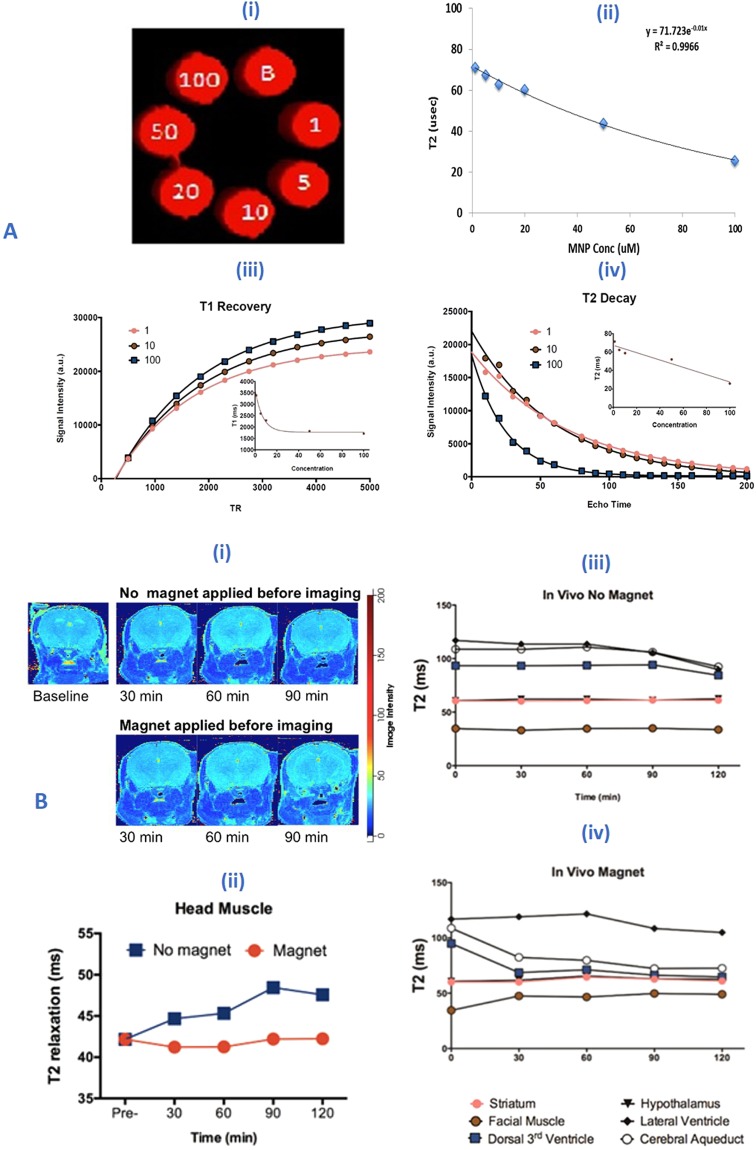
(**A**) *In-vitro* Phantom studies: (i-ii) MRI images of phantoms of MNPs and concentration (0–100 µM) dependent T2 decrease in contrast; iii-iv) T2-decay and T1 recovery images at various concentrations of the MNP suspended in 1% agar solution in different condition i.e. 1, 5, 10, 20 and 100 uM; (**B**) *In-vivo* MRI studies: Data represented NF treated (20 mg/Kg) mice brain after 3 hrs of injection. Changes in T2 relaxation before and after intravenous administration of magneto-liposome NF: (i) T2 relaxation map at baseline, 30–90 minutes post administration; (ii) Average T2 signal intensity from muscle surrounding brain in the head (Magnet condition (0.8 T) received head magnet placement prior to imaging for 3 hours; no magnet condition were without magnet treatment); (iii-iv) T2 signal intensity from different region of brain (different color bullet represents the different location of brain as given below the image).

**Figure 6:**
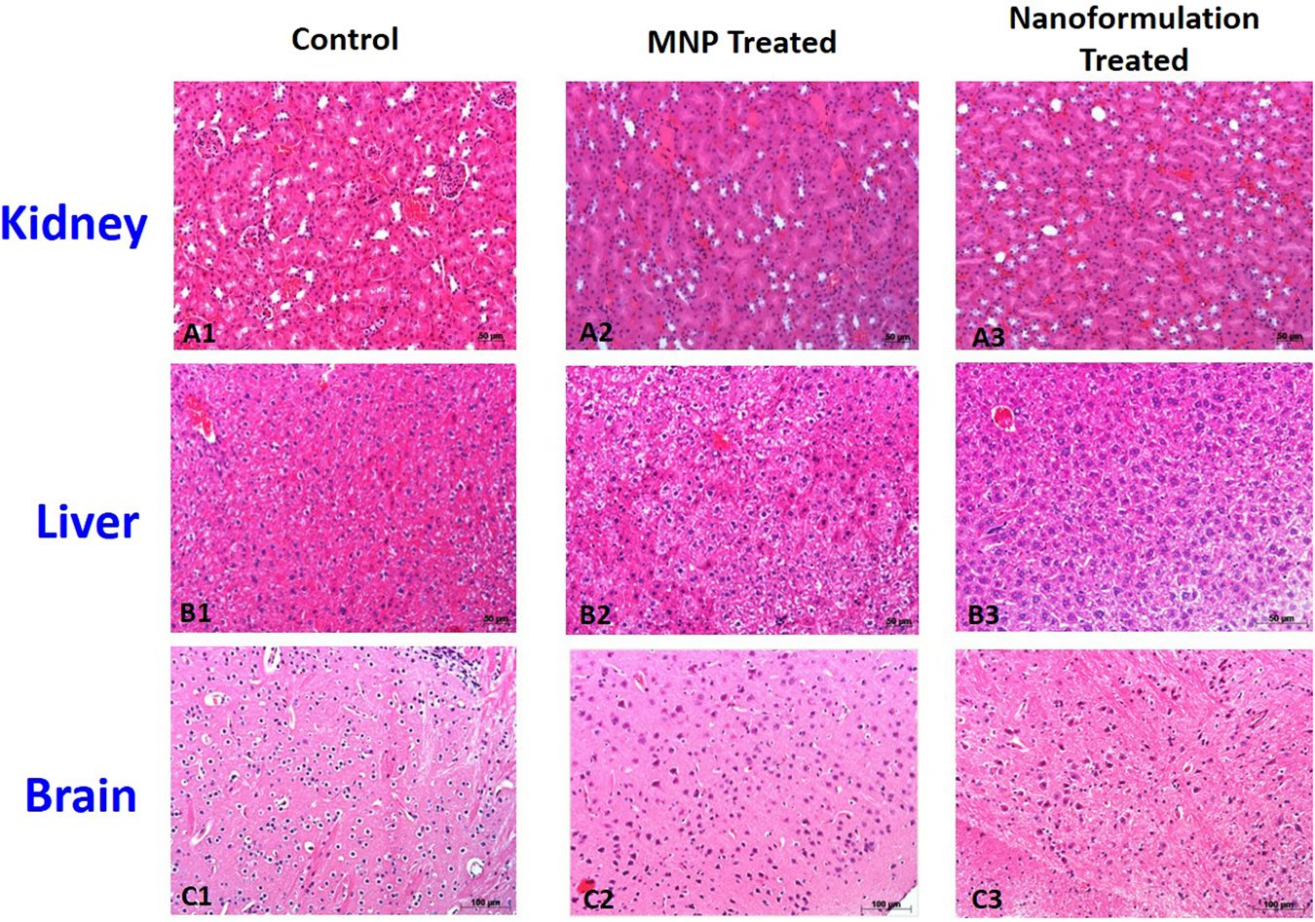
*In-vivo* toxicity study of NF: Representative histopathology of major organs from BALB/C mice treated with of saline (0.9%) for control and 20 mg/Kg (MNP and Nanoformulation) with 0.8 T of an external magnetic field. Kidney (**A1**–**A3**), Liver (**B1**–**B3**), Brain (**C1**–**C3**) were removed from the respective treated animals (1 week) after the injection and analyses for H&E staining (Scale bar = 50–100 µm). No obvious pathological changes were observed in the different tissues.

We confirm relaxivity properties of the paramagnetic MNPs in our phantom studies using 4.7 T. MNPs produced faster T2 relaxation and T1 recovery in a concentration dependent manner (Fig. 5A-i-iv). Further, spin echo T2 image acquisitions revealed no morphological changes or any evidence of tissue damage after NP treatment (Fig. 5B-i). We observed distinct regional and time dependent changes in NF-mediated T2 relaxation, which was dependent on magnetic field driven entry to the brain. At time = 0, T2 values for different tissues at 4.7 T were 40 ms for muscle, 60 ms for brain tissue and >100 ms cerebral spinal fluid. Starting at 30 minutes we observe reduced T2 (from 120 ms to 65 ms) in CSF compartments (cerebral aqueduct and dorsal 3^rd^ ventricle). This is indicative of NF entry in the brain. Changes in T2 were not observed to occur early in mice without magnetic field application prior to imaging. Later in these mice, we observed stable and consistent T2 values across the different tissue compartments (Fig. 5B-ii-iv). Finally, to check the *in-vivo* toxicity of NF, H&E staining was performed to observe any morphological or pathological sign with respect to major organs (i.e. Brain, Liver, and Kidney). The postmortem analysis revealed that there was no evidence of morphological and cytotoxic damage to any of the major organs pertaining to the NF treatment (Fig. 6).

## Discussion

*In-vitro* cell culture based studies and *ex-vivo* studies in HIV-1 infected patients indicate the susceptibility of various CNS cells for the HIV-1 infection. In the brain, macrophages which include perivascular macrophages and resident microglia, productive HIV-1 infection has been reported^28–31^. Although neurons are resistant to HIV infection, neuronal progenitor cells are shown to contain pro-viral DNA^32,33^. While productive HIV-1 infection in astrocytes is debatable, few studies reported restriction of HIV-1 infection and moderately productive HIV-1 infection in astrocytes^34–36^. The evidence of higher levels of HIV-DNA within the 3–19% of a total number of astrocytes with only low productive infection^37–41^ may indicate the HIV latency in these cells, which is a major concern in the elimination of HIV-1 infection from the HIV-infected patients. Numerous *in-vitro* studies have confirmed the persistence of HIV-1 infection in astrocytes^42–47^. These infected astrocytes exposed to the pro-inflammatory cytokines were reported to release the active virus into the cell culture supernatant. Together, these findings show the role of astrocytes and their precursors in establishing the latent/productive HIV-1 infection. The rationale of using the human astrocytes (HA), for this study, is that these cells allow investigation of HIV latency in dividing and differentiated cells. This is important, since, the astrocyte population in the brain consists of cells at different developmental stages and with different mitotic potentials. In this study, we report for the first time, the development of an *in-vitro* model of astrocytes for HIV-1 latency and study the effect of LRA and ARV for purging HIV-1 from CNS reservoirs.

In the case of drug abusing people living with HIV/AIDS (PLWHA), nonadherence to the HAART treatment is a major concern along with the increased incidence of HIV-1 infection and neurocognitive impairment in these patients. Another hurdle in eliminating the HIV-1 infection from the patients and necessity for life-long HAART therapy, is the establishment of latent HIV infection in various parts of the body, especially in the brain, where there is a poor availability of the anti-HIV drugs across the BBB. Molecular mechanisms to repress integrated HIV-1 during latency has been investigated using different models of HIV-1 latency and involvement of multiple mechanisms have been demonstrated. But there are no studies reporting the effect of drugs of abuse in the establishment of the HIV-1 latency and efficacy of the combination of different LRA + ARVs for the complete eradication of HIV-1 from CNS. In this context, depletion of latent HIV-1 reservoirs requires the use of therapeutics targeting both HIV latent cells and active HIV-1 infection. Another issue in CNS targeting is the presence of blood-brain-barrier (BBB), which restricts the transport of most of the ARVs and other potent drugs (e.g. LRA or drug abuse antagonist). Considering this scenario, there is a need to develop an innovative way to overcome the BBB transmigration issue. Nanotechnology has shown great potential in delivering drugs and targeting specific regions in the brain for different CNS disease treatments. Our group has already shown that anti-HIV drug loaded magnetic nanoparticle under the influence of external magnetic field can transport the anti-HIV drug across the BBB and can achieve the desired antiviral efficacy without inducing any cytotoxicity^6,24,27,48,49^.

Even though nanoparticles have big surface area and show great potential for drug delivery, loading hydrophobic drugs (some of ARV or LRA agents), multiple drugs or different classes of anti-HIV drugs along with high loading amounts of drugs on a single nanoparticle, poses as a big challenge. This is the current obstacle in the progress of long-acting nanoformulations. To overcome the above mentioned problems, scientists have used a hybridization strategy in which nanoparticle formulation is encapsulated in liposomes and this kind of magneto-liposomal nanoformulation will provide dual advantage, as in, first, hydrophobic and hydrophilic drugs can be loaded simultaneously in a single formulation and second, loading of magnetic nanoparticles can be used for brain targeting under the influence of an external magnetic field. Further, to solve the problem of low drug loading or a different class of drugs on the surface of an individual nanoparticle, we have investigated the novel approach of LbL self-assembly technique (i.e. adsorption of alternating layers of drugs or biomolecule on the oppositely charged surface)^6^. Due to LbL technique, we were able to load two different classes of antiHIV drugs (Teno and Nel) onto a single nanoparticle surface with high loading efficiency (as shown in Table 1) without any drug-drug interaction. Furthermore, the polyelectrolyte layer of Dextran sulfate helps in achieving the sustained release of drugs *via* diffusion or hydrolysis of LbL surface. Additionally, encapsulation of these drug loaded MNPs within the hydrophilic core of liposome provides the additional barrier for the drug release in a sustained and sequential manner. The rationale of loading different classes of drugs in the NF is to provide better efficacy *via* targeting different stages of HIV-1, latency breaking *via* multiple LRAs to achieve better HIV-1 quiescence *via* acting at the different cellular and viral factors. Additionally, to subdue the effect of Meth on HIV infectivity and overcome the issues of drug addiction, we have used Meth antagonist in our NF to achieve complete protection against HIV-1 infection in CNS. Finally, to deliver these multiple drugs across BBB, we have used the magnetic properties of our NF. MRI studies show that our proposed strategy of magnetic force to deliver drugs across BBB was successful and NF transmigrated across the BBB. Results showed that nanoparticle acrosses the BBB and deposited at various parts of the brain (as shown in Fig. 5B-iv). Finally, *in-vivo* toxicity results showed no alternation in morphological or pathological factors. Further, biochemical and blood profiles studies show no changes in normal values (Supplementary Fig. S4). To take this developed NF to the clinics, we still need to further optimization with regards to better BBB transmigration, higher % drug loading within the liposome (without increasing overall size of NF < 150 nm), and optimization of magnetic treatment (time v/s field strength) for better and longer therapeutic efficacy. Further, we also want to study the effects of different pathological condition (HIV-1 infection v/s HIV-1+ Meth) using appropriate HIV-1 animal model.

## Conclusion

Our data illustrate the potential use of nanotechnology in HIV-1 elimination in drug abusing population. In order to bring the nanotechnology to the clinical level, we need to perform more *in-vivo* studies for the NF efficacy assessments. As the development of CNS latent humanized HIV-1 animal model still poses a big challenge, our lab is working towards this problem and currently developing the HIV-1 infected humanized CD34+ mouse model. For our future work, we will test the drug release kinetics, PK-PD studies and therapeutic efficiency of the developed NF in the proposed murine model ± Meth treatment.

## Electronic supplementary material

Supplemental Figure
