# Supplementary material for: Multifunctional Nanotherapeutics for the Treatment of neuroAIDS in Drug Abusers
Source: Sci Rep. 2018 Aug 28;8:12991. doi: 10.1038/s41598-018-31285-w (PMC6113246; doi:10.1038/s41598-018-31285-w)
Supplement: Supplementary file 1 — Supplemental Figure [file 41598_2018_31285_MOESM1_ESM.pdf]

SUPPLEMENTARY INFORMATION

Multifunctional Nanotherapeutics for the Treatment of neuroAIDS in Drug Abusers

Rahul Dev Jayant[1]*, Sneham Tiwari[1], Venkata Atluri[1], Ajeet Kaushik[1], Asahi Tomitaka[1], Adriana Yndart[1], Luis Colon-Perez[2], Marcelo Febo[2] and Madhavan Nair[1]*

Supplementary Methods

Synthesis of ultra-small magnetic nanoparticles (MNPs) and characterization: The ultra-small Fe_3O_4 MNPs were synthesized according to the co-precipitation method as per protocol published previously by us (Jayant, et al., 2015) and characterized for size and shape using transmission electron microscopy (TEM). The surface charge measurement also carried out at 25°C using dynamic laser scattering (DLS) (Zetasizer NanoZS, Malvern). The superparamagnetic behavior of synthesized MNP was done using vibrating sample magnetometer (Model 4HF VSM, USA). ARV drugs [Tenofovir-Teno and Nelfinavir-Nel] (1mg/ml) was incubated sequentially with (1mg) MNP in PBS buffer (pH 7.4) for 15 minutes at room temperature. The % binding of drugs to MNPs was determined by estimating the concentration of drugs in unbound fraction (supernatant) of the mixture by the spectrophotometric method at UV absorbance of 240 (Nel) and 260 nm (Teno). The polyelectrolyte used for the layer-by-layer (LbL) self-assembly of drugs on MNPs was dextran sulfate sodium salt (DS- polyanion: 2 mg/mL in 0.15 M NaCl; molecular weight ~40,000, Sigma Aldrich, Cat # 42867). Tenofovir (1 mg/mL in PBS; molecular weight, 287.213 g/mol) and Nelfinavir (1 mg/mL in PBS; molecular weight, 663.89 g/mol) were used as polycation. The LbL self-assembly technique was used to load drugs (Nel and Teno) on the MNP surface as per our previously published protocol[6].

***In-vitro* blood-brain-barrier (BBB) preparation, validation, and transmigration assay:** The *in-vitro* BBB model was developed in a bi-compartmental transwell culture plate (Product # 3415,

corning life sciences, Mexico). The upper chamber of this plate is separated from the lower one by a 10µm thick polycarbonate membrane possessing 3.0µm pores. In a sterile 24-well cell culture plate with the pore density of $2x10^6$ pores/cm^2 and cell growth area of 0.33 cm^2, $2x10^5$ HBMEC and HA were grown to confluency on the upper chamber and underside of lower chamber respectively. To assess the effect of NF on the integrity of the *in-vitro* BBB model, after transmigration assay, paracellular transport of FITC-dextran was measured as previously described by us[6]. Also, intactness *of in-vitro* BBB was determined by measuring the transendothelial electrical resistance (TEER) using Millicell ERS microelectrodes (Millipore). Magneto-liposomal NF was collected from lower chambers after 3 hr of magnetic treatment and % transmigration was analyzed at different time points using ammonium thiocyanate-based photometric assay[6, 24].

Intracellular uptake analysis and quantitative analysis: The HA were treated with different concentrations of FITC tagged MLs nanoformulation (50, 100 and 200 µg/mL). The cells were then harvested at 24 hr after treatment, washed and counted; equal amounts of cells (1×10^6) were aliquoted in 1.5 ml Eppendorf centrifuge tubes in PBS. Cell acquisition was performed using ImageStreamX Imaging Flow Cytometer (Amnis Corporation, Seattle, WA) equipped with INSPIRE software. A 60x magnification was used for all samples. A minimum of 10,000 cells was analyzed for each sample. Data analysis was performed using the IDEAS software (Amnis Corporation). FITC and DAPI were excited with a 100 mW of 488 nm argon laser. FITC and DAPI fluorescence was collected on channel two (505–560 nm) and channel seven (560-595 nm), respectively. Intensity adjusted bright field images were collected on channel one. Bright field area and total fluorescence intensity were calculated using IDEAS software. Data analysis was performed using the IDEAS software (Amnis Corporation). Data were compensated using a

compensation matrix generated using singly stained samples. The compensated data was then gated using the following pattern. First, a focus gate was determined to eliminate cells that were not in the field of focus; second, the focused cells were gated to eliminate doublets and debris. Gated data was used to generate histograms measuring fluorescence intensity (sum of all pixels in an image), median pixel intensity (pixel intensity value separating the brighter half from the less bright half), and max pixel intensity (intensity of the brightest pixels in an image) for each sample. The IDEAS software contains wizards to measure internalization and count spots. For the spot counting wizard, subpopulations of cells with low and high NF numbers were manually identified as truth sets, and the software used these data sets to determine the number of FITC-NF spots per cell. The data were reported as a histogram of spots for each sample, and the median spot number was reported. Percent uptake was calculated by multiplying the number of spots per cell by the total cell number. This value (total number of NF taken up by cells) was then divided by the amount of NF added to the cells (which was based on the concentration determination from the ImageStreamX).

MRI and Image analysis: During imaging sessions, mice were anesthetized under 1.5% isoflurane gas, placed in a body tube cradle and setup in a surface transmit/receive radio frequency coil system used for high-resolution imaging on a Magnex Scientific 4.7 Tesla MR scanner. T2 and T1 relaxometry pulse sequences were run on a Varian VnmrJ 3.1 console. Respiratory rates and core body temperature were monitored continuously throughout the experiments. Mice were first scanned without MNP treatment to establish a baseline and were then scanned following intravenous administration of the Magnetic NF. Scanning lasted 6 h, with a scan collected every 30 min. The following parameters were used for T2 relaxation: echo time (TE) = 10-200 at 10 ms

intervals (20 TE's total), repetition time (TR) = 3000 ms, field of view 24 mm^2 along the read, phase directions and 1 mm along the slice direction, and data matrix of 128 x 128 x 10 slices. We also assessed T1 using a saturation recovery sequence with TR (in ms) = 50, 500, 950, 1400, 1850, 2300, 2750, 3200, 3650, 4100, 4550, 5000. Signal averaging was used to increase the signal to noise. Images were imported into NIH Image J (rsbweb.nih.gov/ij) and the QuickVol plugin (Schmidt, et al., 2004) (http://www.quickvol.com) for processing of T2 maps. T2 maps were reconstructed from a non-linear regression of the exponential decay signal using the multi-TE value datasets. T1 maps were also reconstructed with QuickVol using a non-linear regression of the saturation recovery signal. Regions of interest (ROI's) were manually delineated using ITK-SNAP program (Yushkevich, et al., 2006). We selected areas near ventricles (dorsomedial striatum near lateral ventricles, hypothalamus near the 3rd ventricle), and on 3 different ventricular areas (dorsal 3rd ventricle, lateral ventricles, and the cerebral aqueduct). These were clearly visualized on T2 scans. T2 and T1 relaxation rates were assessed separately using MRI on a series of phantom tubes filled with a mixture of Agarose with varying concentrations of magnetic nanoparticle (0-100µM). Parameters similar to those indicated above were used for T2 mapping.

Supplementary Figures

Figure-S1 PMA concentration optimization

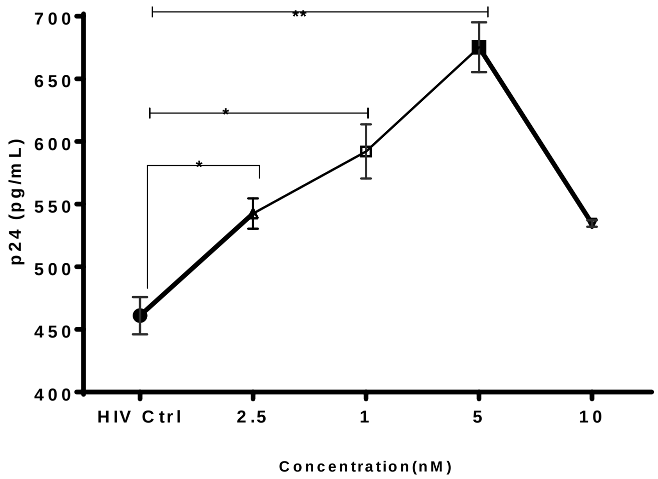

Figure-S2 Meth concentration optimization

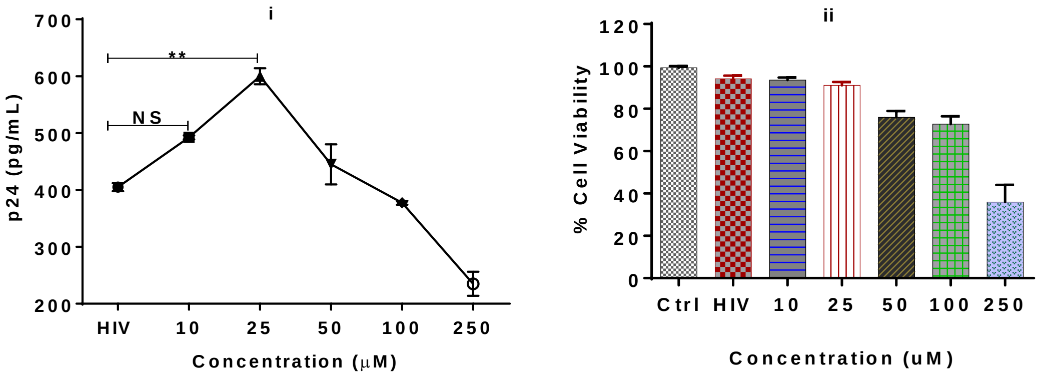

Figure-S3 *In-vitro* cytotoxicity evaluation of different LRA's

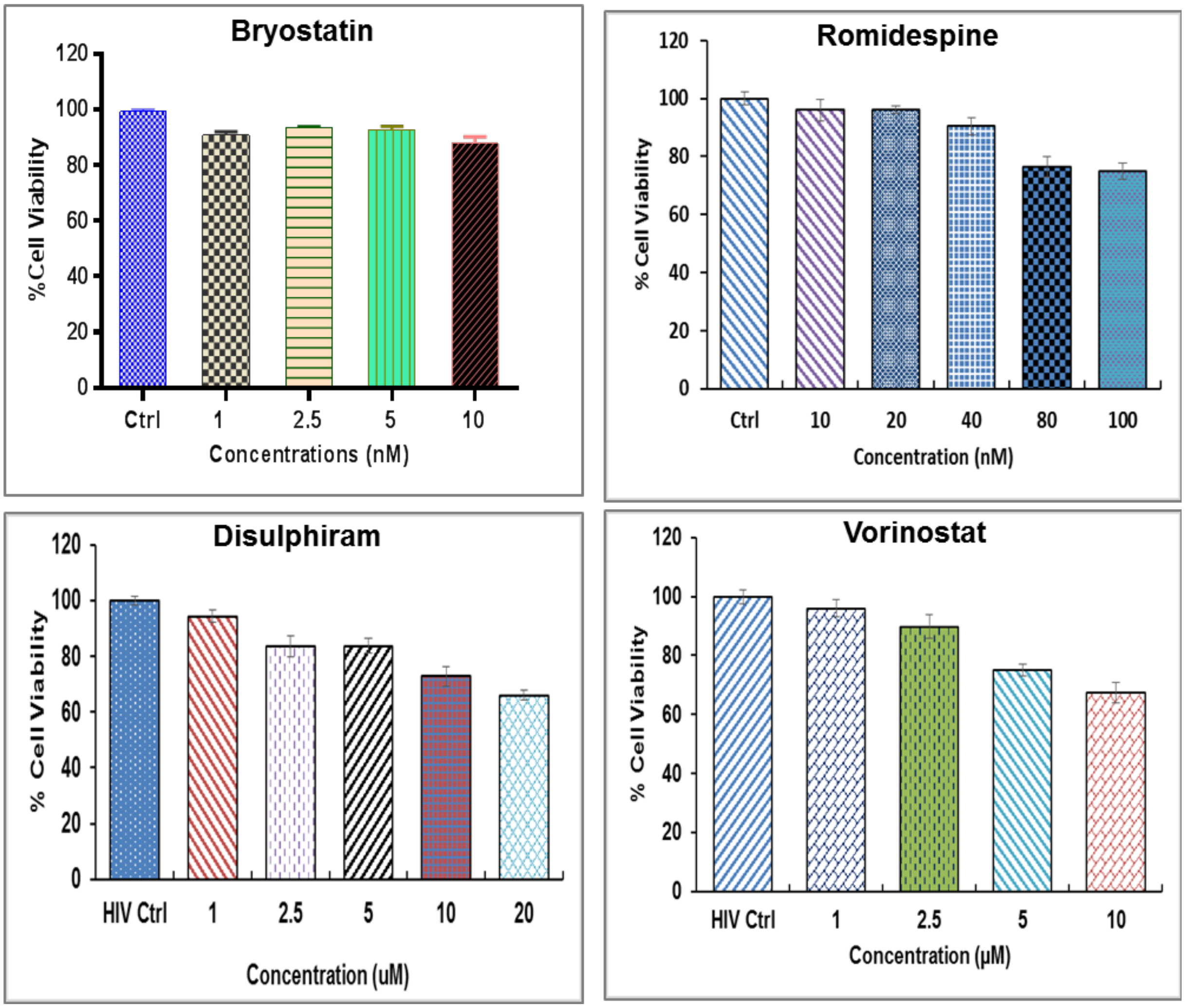

Figure-S4 *In-vivo* blood chemistry profile of MNPs treated and NF treated

Renal/Hepatic Function	Test level			Ref. range
	Control (PBS)	MNP Treated	NF-Treated	
ALBUMIN	3.2 ±0.15	2.8 ±0.19	3.1 ±0.10	2.5-4.6 g/dL
CARBON DIOXIDE	16.9 ±0.45	17.5 ±0.45	16.5 ±0.55	15.0-35.0 mmol/L
CHLORIDE	104.9 ±3.8	100 ±3.2	102 ±4.4	81.0-115 mmol/L
CREATININE JAFFE	0.29 ±0.09	0.33 ±0.10	0.35 ±0.10	0.20-0.90 mg/dL
GLUCOSE	186 ±12.48	179 ±17.44	196 ±15.74	140-263 g/dL
PHOSPHORUS	10.8 ±1.1	9.0 ±2.6	11.0 ±1.4	5.5-12.9 mg/dL
POTASSIUM	8.79 ±0.85	11 ±0.49	9 ±0.94	4-10.5 mmol/L
SODIUM	152 ±11.24	156.4 ±4.62	160.4 ±14.26	110-195 mmol/L
BUN	21 ±1.8	24 ±3.5	24 ±3.5	9-33 mg/dL
ALT	33±8.8	29±4.7	31±7.4	17-77 U/L
AST	87±18.5	98±9.86	90±10.69	52-298 U/L
BILIRUBIN TOTAL	0.1±0.02	0.25±0.05	0.15±0.01	0.0-0.9 mg/dL
ALKALINE PHOSPHATASE	103±1.8	121±3.6	119±2.6	35-222 U/L
PROTEIN TOTAL	5.1±1.8	4.3±1.7	3.9±2.4	3.9-6.4 mg/dL
